# Clinical Prediction of Coronavirus Disease-2019: How Accurate Can One Be?

**DOI:** 10.7759/cureus.11936

**Published:** 2020-12-06

**Authors:** Gabriel Aisenberg, Kevin O Hwang

**Affiliations:** 1 Internal Medicine, John P. and Kathrine G. McGovern School of Medicine at University of Texas, Houston, USA

**Keywords:** covid-19, diagnosis, bias

## Abstract

Background

Some models based on clinical information have been reported to predict which patients have Coronavirus Disease-2019 (COVID-19) pneumonia but have failed so far to yield reliable results. We aimed to determine if physicians were able to accurately predict which patients, as described in clinical vignettes, had, or did not have this infection using their clinical acumen and epidemiological data.

Methods

Of 1177 patients under investigation for COVID-19 admitted, we selected 20 and presented them in a vignette form. We surveyed physicians from different levels of training (<5, and five or more years after graduation from medical school) and included non-medical participants as a control group. We asked all participants to predict the result of the PCR test for COVID-19. We measured the accuracy of responses as a whole, and at three stages of the pandemic associated with a growing incidence of COVID-19 in the community. We calculated the inter-rater reliability, sensitivity, and specificity of the clinical prediction as a whole and by pandemic stage.

Results

Between June 8 and August 28, 2020, 82 doctors and 20 non-medical participants completed the survey. The accuracy was 58% (59% for doctors and 52% for non-medical, p=0.002). The lowest accuracy was noted for cases in the pandemic middle stage; years of post-graduate training represented no difference. Of the 2040 total answers, 1176 were accurate and 864 inaccurate (349 false positives and 515 false negatives).

Conclusion

The influence of symptomatic positivity, confirmation bias, and rapid expertise acquisition on accuracy is discussed, as the disease is new, time after graduation made no difference in the response accuracy. The limited clinical diagnostic capacity emphasizes the need for a reliable diagnostic test.

## Introduction

The diagnostic process for every disease carries inherent difficulties attributable both to the disease itself and to the physician’s skills [1]. This becomes particularly challenging for a new disease with a paucity of clinical information. The clinical presentation, recognition of the underlying pathophysiology, and epidemiological context are the elements required for estimating the pre-test probability of any disease. The test’s positive and negative likelihood ratios will yield a post-test probability to rule in or out the disease, respectively [2].

Different models have been reported to predict which patients are infected with SARS-CoV-2, the causative agent of Coronavirus Disease-2019 (COVID-19), but have failed so far to yield reliable results [3]. Our study aims to determine if, at different moments of the COVID-19 pandemic in Houston, Texas, physicians were able to accurately predict which patients, as described in clinical vignettes, had or did not have this infection using their clinical acumen and epidemiological data.

## Materials and methods

The study was approved by the University of Texas at Houston Committee for the Protection of Human Subjects (HSC-MS-20-0479). Data from de-identified surveys were collected. Written informed consent for this study was waived.

**Figure 1:**
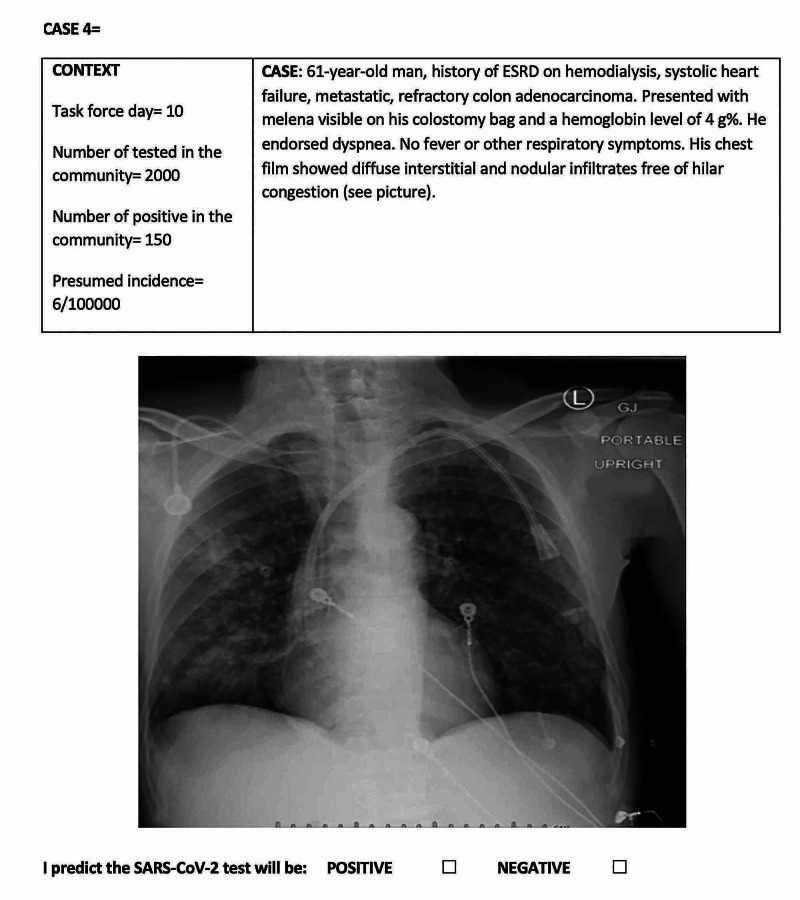
Example of vignette ESRD- End-Stage Renal Disease

On March 12, 2020, our hospital created a task force dedicated to the care of patients with COVID-19 and patients awaiting the result of the SARS-CoV-2 test (person under investigation (PUI)). This clinical suspicion has changed upon frequently updated recommendations by the Centers for Disease Control (CDC). Rapid and accurate testing was not yet available at this early stage of the pandemic. The four task force members, physicians more than five years out from post-graduate training, would informally discuss and try to predict the result of the SARS-CoV-2 test for every PUI admitted to the hospital since meaningful decisions (allocation, treatments, isolation) would result from this assessment. Cases with respiratory symptoms (cough, dyspnea) that presented some degree of difficulty for the prediction of the result of the diagnostic test were deemed appropriate for our survey. Between March 12 and June 15, 2020, 1177 patients were admitted as PUI, of which 255 tested positive for COVID-19. From the list of PUI, we selected 20 patients with whom we created short clinical vignettes including epidemiological data (task force day, number of patients tested in the community, and the incidence as reported daily by the Harris County health authority), as well as clinical information (symptoms, physical exam, laboratory and imaging tests). An example is provided in Figure 1.

We divided the test-cases into three groups based on the time of the pandemic: early cases were those admitted during the first 10 days of the COVID-19 task force when the community incidence was 6 cases/100,000 people. Middle cases were those admitted from day 11 to day 50 of the task force (incidence 137 cases/100,000). Late cases were considered from day 51 to day 80 of the task force (incidence 249 cases/100,000). Because COVID-19 is a new disease, we hypothesized that physicians’ diagnostic accuracy (adequately predicting the positive and negative results) would be high at the beginning of the pandemic and would drop as the incidence grew, resulting from overdiagnosis. We stratified the responses by post-graduate year (less than and five or more years after graduation from medical school) and by speciality (Emergency Medicine (EM), Infectious Diseases (ID), Internal Medicine (IM), and Pulmonary and Critical Care Medicine (PCCM)). We also invited college-educated participants who do not work in the medical field -acquaintances of the authors- as a control group (non-Medical (NM)); we provided this group with no specific training on COVID-19, but only gave them directions on how to fill the survey. We invited each participant to individually review all 20 vignettes and asked them to predict the result of the SARS-CoV-2 test (nasopharyngeal sample, RT-PCR), assuming that every positive would be a true-positive and every negative a true-negative. The cases were emailed to the participants as a single document. Finally, we calculated the sensitivity and specificity of the answers using the RT-PCR as the diagnostic gold standard.

Statistical analysis

MedCalc® version 12.3.0 (MedCalc Software; Mariakerke, Belgium) was used for the statistical analysis. Interrater reliability for all the answers was calculated (Fleiss kappa). Discrete variables (number of correct answers among physicians and NM or physicians graduated less and more than five years after entering the study-were analyzed using the Student t-test for unpaired samples. Single Factor ANOVA was used to compare the accuracy of the responses for early, middle, and late cases among the NM and the physicians separately. A two-sided p < 0.05 was considered statistically significant.

## Results

One hundred and two participants (82 physicians and 20 NM) completed the survey between June 8 and August 28, 2020. The characteristics of all responders are presented in Table 1. The overall participation of doctors from PCCM, EM, and ID was too low to analyze separately. Thus, all doctors’ surveys were grouped, then divided into a group including those with less than five years after graduation (this group included two faculty) and those with five or more years after graduation.

**Table 1:** Characteristics of 102 participants PGY = Post Graduate Year

Speciality	Years of training after graduation
Internal Medicine	PGY 1-4 = 56 participants PGY5 and more = 13 participants
Infectious Diseases	PGY 1-4 = 1 participant PGY5 and more = 3 participants
Emergency Medicine	PGY 1-4 = 1 participant PGY5 and more = 4 participants
Pulmonary and Critical Care	PGY 1-4 = 1 participant PGY5 and more = 3 participants
Non-medical participants	20 participants

The inter-rater reliability was 0.23 (fair) (0.14 for NM, 0.27 for doctors). The responses to the survey are summarized in Table 2. The overall accuracy for the 102 participants was 58% (59% for doctors and 52% for NM, P=0.002). Contrary to our hypothesis, we observed a significant drop in the percentage of correct answers only for the middle cases, with a steep recovery afterwards. Doctors led this curve. The non-medical group showed the same distribution, but the difference between the response accuracy at different pandemic stages was not significant.

**Table 2:** Correct responses (mean± standard deviation) NM= non-medical (*) Difference within the groups at different pandemic stages

	Early cases (6 cases)	Middle cases (9 cases)	Late cases (5 cases)	ALL (20 cases)	p-value* (ANOVA)
All participants n=102	4.02±1.29 (67%)	4.02±1.45 (45%)	3.47±0.82 (69%)	11.51±1.94 (58%)	<0.001
NM n=20	3.55±1.67 (59%)	4.00±1.65 (44%)	2.75±0.72 (55%)	10.30±1.59 (52%)	0.08
Doctors n=82	4.13±1.16 (69%)	4.02±1.41 (45%)	3.65±0.74 (73%)	11.80±1.91 (59%)	<0.001

Of the 2040 total answers (102 participants x 20 answers) 1176 were right, and 864 were wrong (349 false positives and 515 false negatives). An analysis of the answers is presented in Table 3. We found a wide variety of sensitivity and specificity along different pandemic stages.

**Table 3:** Answer accuracy (n=2040)

Prediction of the test result		Result of the SARS-CoV-2 PCR test		Sensitivity	Specificity
		Positive	Negative		
ALL (n=2040)	Positive	505 (25%)	349 (17%)	50%	63%
	Negative	515 (25%)	671 (33%)		
Early (n=612)	Positive	38 (6%)	138 (23%)	37%	73%
	Negative	64 (10%)	372 (61%)		
Middle (n=918)	Positive	217 (24%)	112 (12%)	35%	63%
	Negative	395 (43%)	194 (21%)		
Late (n=510)	Positive	250 (49%)	99 (19%)	82%	51%
	Negative	56 (11%)	105 (21%)		

There was no significant difference in the number and percentage of accurate answers for doctors with less than and five or more years after graduation from medical school: 11.68±1.77 (58%) vs 12.13±2.26 (61%), p=0.34. Both groups continued to show the same accuracy curve as the pandemic advanced (66%, 44%, and 74% correct answers for early, middle, and late cases for doctors up to five years after graduation, p<0.001; and 75%, 47%, and 73% correct answers for those five and more years after graduation, p<0.001).

## Discussion

We demonstrated that physicians’ clinical diagnostic accuracy of COVID-19 dropped as early as in the first 10-50 days but recovered after that regardless of the post-graduate year. The observed errors were both type I (false positive) and II (false negative) evenly.

The drop in accuracy as early as 10 days into the creation of our hospital task force was surprisingly early, considering that at the time, the known incidence in the community was still low. Asymptomatic positivity and confirmation bias could have accounted for this observation [4]. When confirmation bias is at play, the provider's order tests to confirm what they think the patients have: in the chaotic scenario of a pandemic, thinking of a highly incident disease seems plausible for every case, even with a minimal resemblance with the disease at stake [5]. On the other hand, we believe that a higher incidence in the community and expertise-acquisition explain the accuracy recovery for the latest cases. Expertise, questioned in the world of evidence-based medicine, is still necessary to apply a rapidly accumulating body of external evidence [6].

Others have used complex databases including comorbid conditions, medications, exposure to people with COVID-19, to predict COVID-19 with good performance at least in the geographic area of validation (Florida and Ohio) [7]. While the presumptive diagnosis of COVID-19 relies on clinical and radiological data, only the RT-PCR of oropharyngeal samples or related methods can confirm the diagnosis [8,9].

Interestingly, in our study, we found no difference in accuracy related to the year of graduation. This is likely because, regardless of the medical experience, the disease was new to every participant.

Limitations

First, the clinical COVID-19 case definition provided by the Centers for Disease Control and Prevention has changed while we were selecting cases [10]; nevertheless, the last update occurred before we started collecting surveys. Second, our testing capability changed along the case selection period, leading to more testing, even among patients with presumed low-risk. At the same time, this reflects a real-world scenario; it also gave us less typical cases to select from leading to a greater difficulty at predicting the result of the test. Third, the definition of early, middle, and late periods are arbitrary based on the increased incidence in the community; there was no prior published paper guiding this decision; moreover, whether a selection of later study cases when the incidence was even higher or the selection of more typical cases (both for predictive positive and negative) would have modified the results is unclear. Fourth, while the cases might have been chosen at different times of the pandemic, the participants answered after all the cases had been selected; therefore, to understand the influence of the changing incidence of COVID-19 on the expected result of the test, they would have had to overcome their perception of the incidence at the time of completing the survey. Furthermore, even for diseases other than COVID-19, studies have shown that the influence of epidemiological information on diagnosis is frequently inaccurately interpreted by physicians [11]. Finally, the low participation of specialists in EM, ID, and PCCM prevented us from understanding how these specialists would have answered. 

## Conclusions

To accurately diagnose a new disease, several factors must converge: a clear definition of the case, a reliable diagnostic test, and expertise to interpret the information. Physicians should remain humble and aware of the fact that when the disease is new, the ability to diagnose it is expected to change as new information solidifies our knowledge.
